# Quality of life in patients with dysphagia after radiation and chemotherapy treatment for head and neck tumors

**DOI:** 10.4317/jced.51092

**Published:** 2013-07-01

**Authors:** Renata JDS. de Campos, Pamella V. Palma, Isabel CG. Leite

**Affiliations:** 1 Oncology Institute of Juiz de Fora; Voice specialist; Master Program of Brazilian Health, Federal University of Juiz de Fora, Minas Gerais, Brazil; 2 Graduate student at the School of Dentistry, Institutional Programs for Scientific Start-up Grants (XX PIBIC/UFJF). Federal University of Juiz de Fora – UFJF, Brazil; 3 Doctor Professor, College of Medicine, Federal University of Juiz de Fora - UFJF - Juiz de Fora, MG, Brazil

## Abstract

Objective: The aim of this study is to analyze subjectively, using the SWAL-QOL questionnaire, swallowing dysfunction and associated factors after treatment with radiotherapy and chemotherapy in patients treated for head and neck cancer. Material and Methods: This is a cross-sectional study, based on the selection of patients with tumors of the head and neck area, treated with radiotherapy with or without chemotherapy during the years 2000 to 2006 at the Oncology Institute of Juiz de Fora. The data were analyzed using SPSS 15.0 software, and were evaluated using the chi-square test to compare differences in proportions between groups. The statistical significance level was set at 5%. Results: It was observed that with respect to foods of solid consistency, there was a statistically significant difference for mouth tumors (p<0.01), with a tendency in this group to use softer foods, easier to chew (stews, boiled vegetables, creamy soups, canned fruit). With reference to the domains of the SWAL-QOL, the location of the tumor in the mouth was statistically associated with the lowest quality of life in the symptoms domain (p<0.05). The female gender variable was associated with the lowest perceived quality of life in several domains, namely swallowing (p=0.02); fatigue (p=0.008); symptoms (p=0.009). Age (split below and above 60 years) was not associated with differences in perceived quality of life in any domain. Conclusion: Tumor in the mouth and the total dose of radiation in the superior fossa were associated with the lowest quality of life in the symptoms domain. The female gender variable was associated with the lowest perceived quality of life in several domains This study shows that speech therapy should maintain a presence in the teams, to then guide the rehabilitation of organic dysphonia and mechanical dysphagia possibly afflicting patients after cancer treatment with radiation therapy and chemotherapy.

** Key words:**Quality of life, dysphagia, head and neck neoplasms, rehabilitation.

## Introduction

Treatment for head and neck cancer aims to cure the disease, along with good functional results in both speech and swallowing (1). The preferred method of treatment varies depending on the tumor site, size, and presence or absence of regional lymph nodes. The combination of radiotherapy and chemotherapy promotes preservation of the structures maintaining proper function, and it turns out that since the 1980s, the number of articles published in which authors claim that organ preservation, in many cases, may be equivalent to the preservation of function, is becoming ever greater.

Functional results of the combination of radiotherapy and chemotherapy for tumors in the head and neck area can preserve the organ and work to maintain functionality (2-6). However, radiotherapy can cause other disorders in different degrees of severity, notably: edema and fibrosis of the exposed region, trismus, mucositis, xerostomia, odynophagia, actinic dermatitis, substantial weight loss, and the need to use alternative feeding routes (7).

Impairment in the efficiency of swallowing or in the safety of swallowing may afflict patients treated with organ preserving procedures, which may exhibit different consequences depending on the changes occurring in the treatment due to anatomical and functional changes (8,9).

The SWAL-QOL is an important tool for assessing the patient’s point of view on the effectiveness of rehabilitation, as regards the impact of impairment of swallowing on quality of life (10,11).

This work is justified by the importance of the occurrence of swallowing problems after radiotherapy and / or chemotherapy treatment for cancer of the head and neck. These significant impairments in patients’ oral com-munication interfere with their quality of life. Moreover, treatment directed at these vocal disorders is low cost and provides significant improvement in the quality of life and voice of these patients.

## Material and Methods

The study was conducted in the city of Juiz de Fora, located in the Zona da Mata region in southeastern Minas Gerais, and identified as the fourth most populous municipality in the state. It is the main industrial, cultural, and educational center of the Zona da Mata of Minas Gerais, especially in relation to health care, and can be considered a major hub for health services in the region.

The study sample was designed in the form of a cross-sectional study, based on the selection of cases diagnosed and treated in the Radiotherapy Section of the Oncology Institute of Juiz de Fora, from the hospital records of the institution. It was approved by the Ethics Committee on Human Research of PROPESQ / UFJF under protocol no. 236/2007.

The inclusion criteria for patient admission of in the study were patients with tumors of the oral cavity, pharynx, and larynx, diagnosed and treated in the aforementioned hospital, in the period from 2000 to 2006, with stage T1, T2, T3, or T4 (in the latter case, for patients who refused surgical treatment); and with a proposed treatment of radiotherapy, exclusively or concomitant with chemotherapy, with a curative intent, through cervicofacial fields, augmented or not from the supraclavicular fossa field, or a proposed palliative therapy; and also patients who did speech therapy prior to, during, or after such medical treatments. The exclusion criteria were patients that had died during the study period; patients who underwent surgical treatment in the head and neck area due to cancer, combined or not with other procedures; a progressive history of neurological disorders, due to possible interference in larynx physiology; as well as impairments of a physical, motor, and / or emotional nature that prevented the patient from participating in the study. They were diagnosed in oncology. All treatment was done in another institution which may influence the quality of life and differentiate them from other patients.

Out of 698 patients identified as receiving care between 2000 and 2006 for the treatment of tumors of the oral cavity, pharynx and larynx, 394 were excluded from the study because hospital records showed surgical treat-ment for cancer, and 304 patients, who had undergone radiotherapy and / or chemotherapy for the treatment of head and neck cancer, were included in the study. Following hospital records analysis and sample identification, contacts were made by phone and it was determined that 79 patients had died; 150 had outdated records, making contact impossible; 35 had undergone salvage surgery; 2 had undergone chemotherapy only; 7 patients refused to participate in the research; and 6 were not treated at the Institute. So, ultimately, 25 patients were evaluated.

The 25 patients were located by the Speech Therapy Service of the Oncology Institute of Juiz de Fora, and invited to participate in the research. After they signed free and informed consent statements, their research questionnaire responses and voice measurements were taken.

The study method consisted of applying the Quality of Life in Swallowing Disorders (SWAL-QOL) question-naire to patients after treatment with radiotherapy and / or chemotherapy for head and neck tumors, independent of swallowing dysfunction complaints (12), and validated in Portuguese (13). SWAL-QOL is divided into 44 questions that assess eleven domains, those being: swallowing as a burden, desire to eat, meal duration, frequency of symptoms, food selection, communication, fear of eating, mental health, social life, sleep, and fatigue.

The data were analyzed with SPSS 15.0 software, and were evaluated using the chi-square test, to compare differences in proportions between the analysis groups (according to anatomic location, gender, therapeutic intervention standard). For continuous variables, means were analyzed using the Student’s t-test. The comparison of ordinal variables (quality of life and voice quality scales) with nominal outcomes (type of treatment, gender, tumor location) was performed using the Kruskal-Wallis test. The statistical significance level was set at 5%.

## Results

From the sample, 54% of the group were aged between 51 and 69 years. The self-reported skin color was white in 72% of the cases. Gender was male in 72% of the cases. The predominant education level was “up to 8 years” in 56% of the patients.

As for the tumor and treatment characteristics ( Table 1) we observed 60% of the tumors were laryngeal, stage II being the most common, accounting for 32%. The therapeutic procedure used most often was radiotherapy and chemotherapy.

**Table 1:**
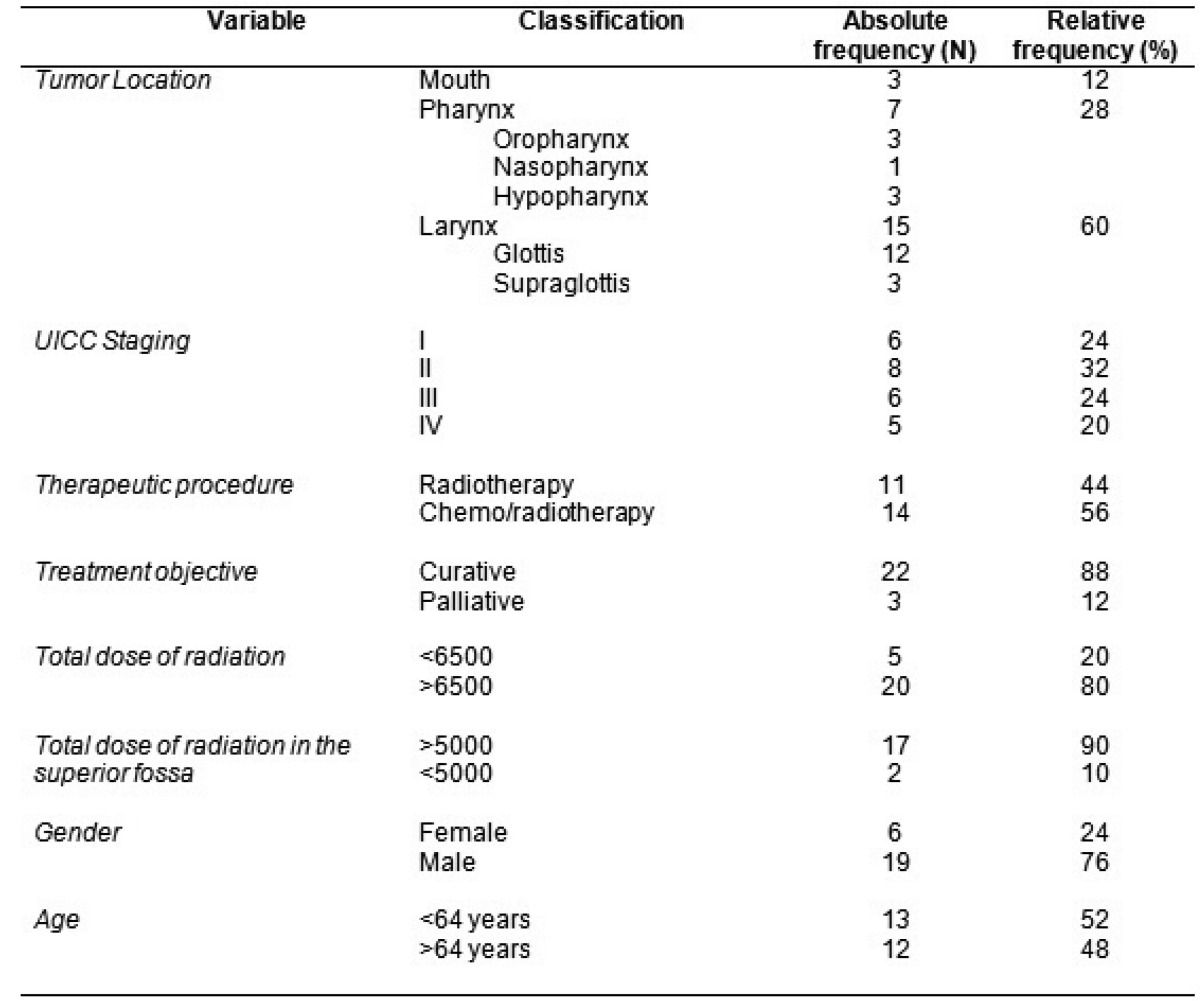
Characteristics of the tumor and treatment implemented and sociodemographic profile of patients with cancer of the head and neck treated using organ-preserving procedures, Brazil, 2011.

At the time of the interview, none of the patients were making use of feeding tubes and consuming liquid food in any consistency exclusively. Regarding the consistency of solid foods, there was a statistically significant difference for mouth tumors (p<0.01), with a tendency in this group to use softer foods, easier to chew (stews, boiled vegetables, creamy soups, canned fruit).

With reference to the SWAL-QOL domains, the location of the tumor in the mouth was statistically associated with the lowest quality of life in the symptoms domain (p<0.05). The total dose of radiation in the superior fossa above 5000 Gy also stood out in the symptoms domain with a worse score (p=0.08), and total radiation received above 7000 Gy was associated with worse scores in the sleep domain (p=0.008). The female gender variable was associated with the lowest perceived quality of life in several domains, namely swallowing (p=0.02); fatigue (p=0.008); symptoms (p=0.009). Age (split below and above 60 years) was not associated with differences in perceived of quality of life in any domain.

In the health self-assessment, there were no differences in relation to anatomical location, nor gender.

 Table 2 and  Table 3 show the SWAL-QOL results observed in the patients studied.

**Table 2:**
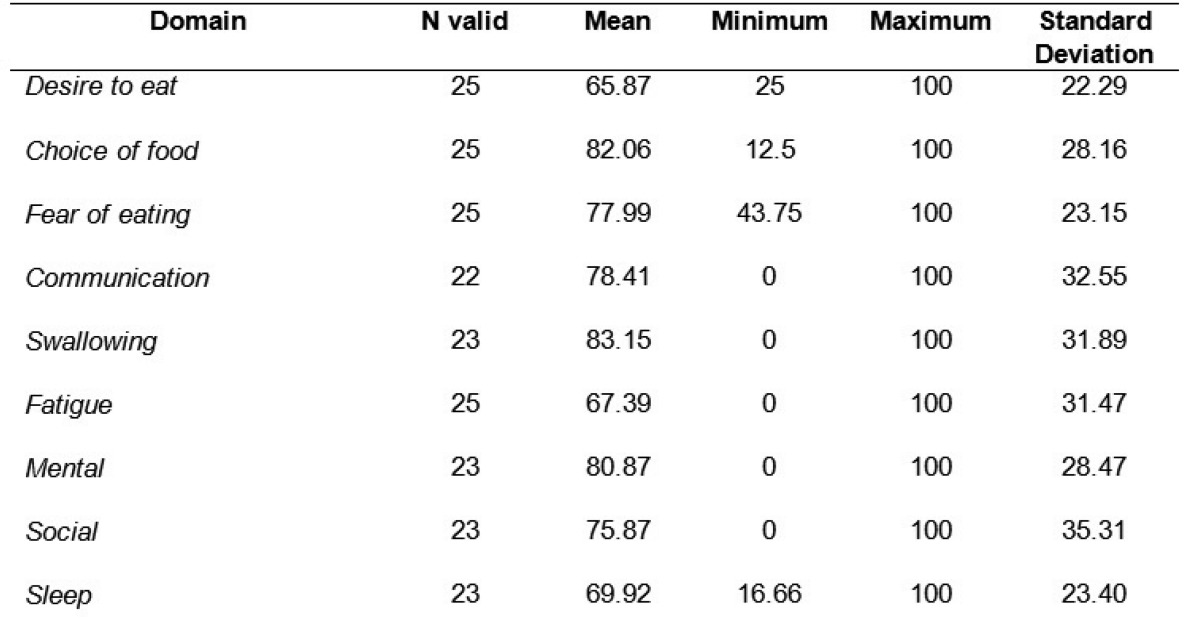
Mean scores for the SWAL-QOL domeins, from patients with head and neck cancer treated using organ-preserving procedures, Brazil, 2011.

**Table 3:**
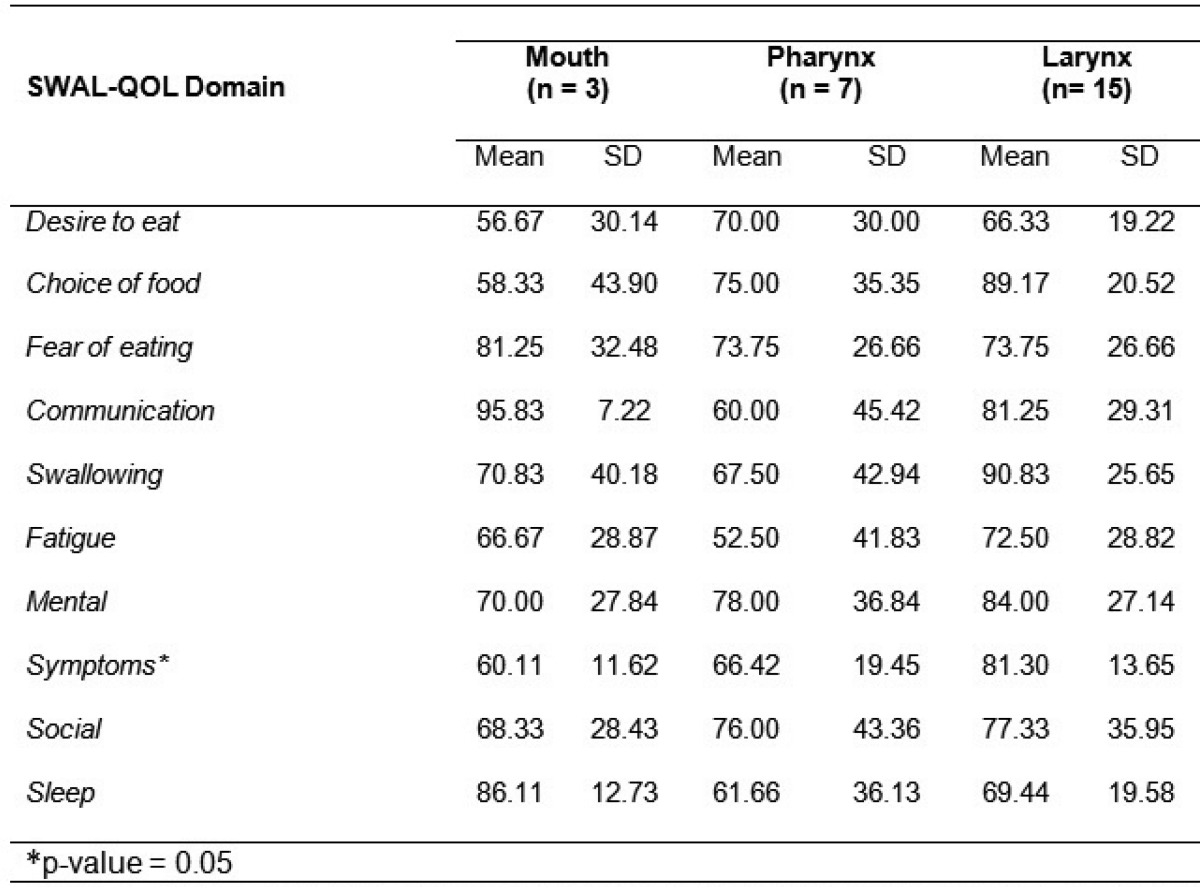
Mean scores and standard deviations for the SWAL-SOL domains, according to anatomical location of the tumor, Brazil, 2011.

## Discussion

The SWAL-QOL questionnaire is described as an important tool for researchers to assess quality of life and dysphagia. It consists of direct and practical questions that evaluate the quality of life of patients, who for any given treatment, exhibit dysphagia as a consequence (12).

A study on patients after treatment for nasopharyngeal carcinoma was developed using the University of Washington (UW-QOL) questionnaire and the SWAL-QOL. As a result, 43 patients self-reported difficulty in swallowing. The study concluded that swallowing difficulty could negatively influence the quality of life of patients after treatment for nasopharyngeal carcinoma, and that the application of a specific questionnaire to measure this problem, such as SWAL-QOL, is becoming necessary, as well as more research in this area to determine parameters for delivering better treatment to patients (14).

A study aiming to describe the quality of life related to swallowing, in patients treated for tongue cancer, was developed, with 29 patients participating in the study. Patients with an advanced disease level, who underwent radiation therapy for tumor treatment, had worse scores on most of the domains, such as problems chewing, pleasure in eating, among others (15).

The feasibility, reliability, and validity of the SWAL-QOL questionnaire was evaluated, and as a result obtained high reliability, good feasibility, and except for patients with a feeding tube, validity for patients with swallowing problems. The group suggests further comparative studies by applying this questionnaire for different tumor locations, and as an instrument capable of identifying the need for speech rehabilitation therapy among patients who present difficulty in swallowing (16).

The SWAL-QOL questionnaire for assessing quality of life was validated for the French language, results being collected from patients after surgical treatment of tumors of the oropharynx, and patients that had suffered a stroke. The French version is considered understandable and acceptable by patients, helping to evaluate the quality of life of patients with oropharyngeal dysphagia, and contributing to their referral for speech rehabilitation therapy (17).

Quality of life is an important measure of health status and the effects of therapy in patients with oropharyngeal dysphagia. The validity of the SWAL-QOL was evaluated, and in this study singled out as the gold standard or reference, when compared with two other questionnaires that measure quality of life (18). The SWAL-QOL questionnaire obtained a high level of patient acceptance, with excellent psychometric properties, in a study on its validation for the Swedish language (19).

The aftermath of this medical treatment turns out directly affecting oral communication, voice, and swallowing for the patients treated, thus speech therapy should maintain a presence in the teams, to then guide the rehabilitation of organic dysphonia and mechanical dysphagia possibly afflicting patients after cancer treatment with radiotherapy and chemotherapy.

Speech therapy is aimed at training and at rehabilitation of oral communication, voice, and swallowing disorders that can afflict patients after radiotherapy and chemotherapy for head and neck cancers. The oncological rehabilitation, proposed by the speech therapist, restores the functions of speech, voice, and swallowing in patients who have undergone treatment for head and neck tumors. It is becoming important to publish research that determines the disorders most common for these patients, and their impact on patient quality of life, so that techniques can be established that enable a better restoration of these functions that directly affect the daily life of the patient.
